# Dysthymia and Apathy: Diagnosis and Treatment

**DOI:** 10.1155/2011/893905

**Published:** 2011-06-27

**Authors:** Junko Ishizaki, Masaru Mimura

**Affiliations:** ^1^Department of Psychiatry, Nagata Hospital, 5173 Goji-cho, Miyakonojo-shi, Miyazaki 885-0084, Japan; ^2^Department of Neuropsychiatry, Showa University School of Medicine, 6-11-11 Kita-Karasuyama, Setagaya-ku, Tokyo 157-8577, Japan; ^3^Department of Neuropsychiatry, Keio University School of Medicine, 35 Shinanomachi, Shinjuku-ku, Tokyo 160-8582, Japan

## Abstract

Dysthymia is a depressive mood disorder characterized by chronic and persistent but mild depression. It is often difficult to be distinguished from major depression, specifically in its partially remitted state because “loss of interest” or “apathy” tends to prevail both in dysthymia, and remitted depression. Apathy may also occur in various psychiatric and neurological disorders, including schizophrenia, stroke, Parkinson's disease, progressive supranuclear palsy, Huntington's disease, and dementias such as Alzheimer's disease, vascular dementia, and frontotemporal dementia. It is symptomatologically important that apathy is related to, but different from, major depression from the viewpoint of its causes and treatment. Antidepressants, especially noradrenergic agents, are useful for depression-related apathy. However, selective serotonin reuptake inhibitors (SSRIs) may be less effective for apathy in depressed elderly patients and have even been reported to worsen apathy. Dopaminergic agonists seem to be effective for apathy. Acetylcholine esterase inhibitors, methylphenidate, atypical antipsychotics, nicergoline, and cilostazol are another choice. Medication choice should be determined according to the background and underlying etiology of the targeting disease.

## 1. Dysthymia

Dysthymia is a depressive mood disorder that is characterized by chronic, persistent but mild depression, affecting 3–6% of individuals in the community [1, 2] and as many as 36% of outpatients in mental health settings [3]. Although by definition, the depressed mood of dysthymia is not severe enough to meet the criteria for major depressive disorder, it is accompanied by significant subjective distress or impairment of social, occupational, or other important activities as a result of mood disturbance [4]. Dysthymia manifests as a depressed mood persisting for at least two years (one year for children or adolescents) that lasts for most of the day, occurs on more days than not, and is accompanied by at least two of the following symptoms:

1. poor appetite or overeating,
2. insomnia or hypersomnia,
3. low energy or fatigue,
4. low self-esteem,
5. poor concentration or difficulty making decisions,
6. feelings of hopelessness.

To diagnose dysthymia, major depressive episodes must not have occurred during the first two years of the illness (one year in children or adolescents), and there should be no history of mania. The Diagnostic and Statistical Manual of Mental Disorders, Fourth Edition, Text Revision (DSM-IV-TR) [5] states that transient euthymic episodes lasting for up to two months may occur during the course of dysthymia. In the past, dysthymia has had several other names, including depressive neurosis, neurotic depression, depressive personality disorder, and persistent anxiety depression.

DSM-IV-TR categorizes dysthymia according to several course specifiers: (1) early onset if symptoms begin before the age of 21 years, (2) late onset if symptoms begin at age 21 or later, and (3) dysthymia with atypical features if symptoms include increased appetite or weight gain, hypersomnia, a feeling of leaden paralysis, and extreme sensitivity to rejection.

It is often difficult to differentiate dysthymia from major depression specifically in patients with partial remission or partial response to treatment. Major depressive disorder, dysthymia, double depression, and some apparently transient dysphorias may all be manifestations of the same disease process. These varieties of depressed mood states, while distinct diagnostic entities, share similar symptoms and respond to similar pharmacologic and psychotherapeutic approaches. Due to the stigma still associated with depression, many people with this disorder may be unrecognized and untreated. Although dysthymia has long been considered to be less severe than major depression, the consequences of this condition are increasingly recognized as potentially grave, including severe functional impairment, increased morbidity from physical disease, and even an increased risk of suicide. 

The pathophysiology of dysthymia is not fully understood. Approximately 30% of individuals with dysthymia show a switch to hypomanic episodes at some stage [6]. Most people, especially those with early onset dysthymia, have a family history of mood disorders, including bipolar disorder. One or both parents may have suffered from major depression. A family history of this illness makes it more likely for dysthymia to appear in the teenage years or early 20s. Compared with major depression, patients with dysthymia tend to have more subjective symptoms and less dramatic psychomotor disturbance or neurovegetative symptoms including abnormalities of sleep, appetite, and energy levels. A longitudinal prospective study revealed that 76% of dysthymic children develop major depression, and 13% develop bipolar disorder over follow-up periods of 3–12 years [7]. In the other study, it should be noted that around 75% of people with dysthymia meet the criteria for at least one major depressive episode, and this combination is referred to as double depression [8]. Persons with dysthymia who have major depressive episodes tend to suffer from depression for long periods and spend less time fully recovered [9]. In a 10-year follow-up study of persons with dysthymia, 73.9% showed recovery from dysthymic disorder, with a median time to recovery of 52 months, but the estimated risk of relapse into another period of chronic depression including dysthymia was 71.4%, most commonly within three years [10].

The validity of making a distinction between depressive personality disorder and dysthymia has been a matter of debate since depressive personality disorder and dysthymia are both classified among the lesser severity spectrum of depressive disorders. Depressive personality disorder is characterized by a gloomy or negative outlook on life, introversion, a tendency toward self-criticism, and pessimistic cognitive processes, with fewer than mood and neurovegetative symptoms, seen in dysthymia. Dysthymia or depression may coexist with depressive personality disorder, and persons who have depressive personality disorder are at greater risk of developing dysthymia than healthy persons after followup for 3 years [11].

## 2. Treatment for Dysthymia

The best treatment for dysthymia appears to be a combination of psychotherapy and medication. The positive clinical response to medications like selective serotonin reuptake inhibitors (SSRIs) [12–19], serotonin norepinephrine reuptake inhibitors (SNRIs) [20, 21], and tricyclic antidepressants (TCAs) [14, 15] suggests that serotoninergic and noradrenergic systems involve the mechanism of dysthymia. A systematic review [22, 23] of antidepressant treatment for dysthymia suggests that SSRIs, TCAs, and monoamine oxidase inhibitors are all equally effective, but SSRIs may be slightly better tolerated. Success has also been reported with more noradrenergic agents, such as mirtazapine, nefazodone, venlafaxine, duloxetine, and bupropion. Second-generation antipsychotics showed beneficial effects compared to placebo for major depressive disorder or dysthymia, but most second-generation antipsychotics have shown worse tolerability, mainly due to sedation, weight gain, or laboratory data abnormalities such as prolactin increase. Some evidence indicated beneficial effects of low-dose amisulpride for dysthymic people [24]. 

Psychotherapy and medication are both effective treatment modalities for dysthymia and their use in combination is common. There are many different types of psychotherapy, including cognitive behavioral therapy, psychodynamic, and insight-oriented or interpersonal psychotherapy, which are available to help persons with dysthymia. Cognitive Behavioral Analysis System of Psychotherapy (CBASP) [25] has been attracting more attention for the treatment of chronic depression. CBASP is a form of psychotherapy that was specifically developed for patients with chronic depression. Its core procedure is called “situational analysis” and is a highly structured technique that teaches chronically depressed patients how to handle problematic interpersonal encounters. It encourages patients to focus on the consequences of their behavior and to use a social problem-solving algorithm to address interpersonal difficulties. CBASP is more structured and directive than interpersonal psychotherapy and differs from cognitive therapy by focusing primarily on interpersonal interactions, including interactions with therapists. Through this psychotherapy, patients come to recognize how their cognitive and behavioral patterns produce and perpetuate interpersonal problems and learn how to remedy maladaptive patterns of interpersonal behavior. The combination of medication and psychotherapy may be much more effective than either one alone [26].

## 3. Apathy

Dysthymia is essentially defined by the existence of depressive symptoms at some level. However, some patients who are treated for dysthymia only present with loss of interest and do not have a depressed mood. This condition should be regarded as apathy. The term “apathy” is derived from the Greek “pathos” meaning passion, that is, apathy means “lack of passion”. Marin [27] defined the apathy syndrome as a syndrome of primary lack of motivation, that is, loss of motivation that is not attributable to emotional distress, intellectual impairment, or diminished consciousness. Starkstein [28] described the features of apathy as lack of motivation characterized by diminished goal-oriented behavior and cognition, and a diminished emotional connection to goal-directed behavior. Levy and Dubois [29] proposed that apathy could be defined as the quantitative reduction of self-generated voluntary and purposeful behavior. At present, apathy is treated symptomatically. There is no decision tree for apathy in DSM-IV-TR, but there is a possibility that apathy will come to be managed independently from mood disorders if the mechanisms involved or treatment strategy is more fully established in the future. Marin [27] and Starkstein [30] have suggested diagnostic criteria for this condition. As the basis of specific diagnostic criteria for apathy, abnormalities in aspects of emotion, cognition, motor function, and motivation have been suggested. Marin has also developed an apathy rating scale [31], while diagnostic criteria for apathy have been proposed by Starkstein et al. (Table 1).

**Figure 1:**
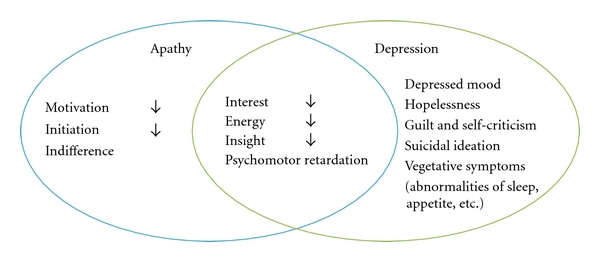
Apathy versus depression.

Apathy has received increasing attention because of its effects on emotion, behavior, and cognitive function. It seems likely that apathy in persons with depression results from alterations of the emotional and affective processing, but it may typically occur in the absence of a depressed mood (Figure 1).

 Apathy occurs in persons with a variety of psychiatric and neurological disorders including schizophrenia [32, 33], stroke [34, 35], traumatic brain injury [36], Parkinson's disease [28, 37, 38], progressive supranuclear palsy [38], Huntington's disease [39, 40], and dementias such as Alzheimer's disease [30, 41, 42], vascular dementia [43], frontotemporal dementia [41, 42], and dementia due to HIV [44]. Marin et al. [45] evaluated five subgroups (healthy elderly adults, patients with left hemispheric stroke, right hemispheric stroke, Alzheimer's disease, and major depression) by using the apathy evaluation scale [31] and the Hamilton rating scale for depression [46]. Mean apathy scores were significantly higher than healthy elderly scores in right hemispheric stroke, Alzheimer's disease, and major depression. Elevated apathy scores were associated with low depression in Alzheimer's disease, high depression in major depression, and intermediate scores for depression in right hemispheric stroke. The prevalence of elevated apathy scores ranged from 73% in Alzheimer's disease, 53% in major depression, 32% in right hemispheric stroke, 22% in left hemispheric stroke, and 7% in normal subjects. They found that the level of apathy and depression varied among diagnostic groups although apathy and depression were significantly correlated within each group. Thus, apathy is most often seen clinically within the setting of depression, dementia, or stroke, and problems related to apathy tend to be important because of its frequency, increasing prevalence, impact on daily life, poorer rehabilitation outcomes after stroke, and burden on caregivers. 

Levy et al. [42, 47] found that patients with frontotemporal dementia and progressive supranuclear palsy could be discriminated from patients with Alzheimer's disease by their more severe apathy and relatively less severe depression. Furthermore, they reported that apathy was not correlated with depression in a combined patient sample, including those with Alzheimer's disease, frontotemporal dementia, progressive supranuclear palsy, Parkinson's disease, and Huntington's disease. Apathy, but not depression, was correlated with lower cognitive function as measured by the mini mental state examination [48]. These results imply that apathy might be a specific neuropsychiatric syndrome that is distinct from depression but is associated with both depression and dementia. Symptomatologically, it is important to understand that apathy can occur concomitantly with depression, but is usually different from it. Depression is a “disorder of emotion”, while apathy is a “disorder of motivation”. Starkstein et al. [34] studied the frequency of apathy among stroke patients with major depression, minor depression, or no depression. A fairly large number (23%) of their patients had significant apathy. The apathetic patients were older, had a higher frequency of major (but not minor) depression, had more severe physical and cognitive impairment, and had lesions involving the posterior limb of the internal capsule. In their study, there was a significantly higher frequency of apathy among the patients with major depression but not those with minor depression or no depression. These findings indicate that although major depression and apathy occur independently, apathy remains significantly associated with major depression (but not with minor depression). This is consistent with the results of previous studies that have differentiated between major and minor depression, including differences of cognitive function and cortisol suppression after dexamethasone administration [49, 50], which were seen in patients with major depression but not minor depression. 

Apathy is often seen in patients with lesions of the prefrontal cortex [51, 52] and is also frequent after focal lesions of specific structures in the basal ganglia such as the caudate nucleus, the internal pallidum, and the medial dorsal thalamic nuclei [53–56]. Apathy is, therefore, one of the clinical sequelae of disruption of the prefrontal cortex-basal ganglia axis, which is one of the functional systems involved in the origin and control of self-generated purposeful behavior. Anatomical localization of regional dysfunction associated with apathy and depression appears to overlap considerably. Depression has been reported to be more frequent when focal lesions are anterior and left-sided [57]. Levy and Dubois [29] proposed that the mechanisms responsible for apathy could be divided into three subtypes of disrupted processing: “emotional-affective”, “cognitive”, and “autoactivation” loss of psychic self-activation.

## 4. Treatment for Apathy (Table 2)

Taking into consideration the facts that apathy is related to cognitive function and disruption of the prefrontal cortex-basal ganglia axis, apathy can be considered to resemble subcortical dementia and to be treatable using dopaminergic agents in central nervous system. A growing number of reports have documented the treatment of apathy with a variety of psychoactive agents. Various small studies have indicated that psychostimulants, dopaminergics, and cholinesterase inhibitors might be of benefit for this syndrome. However, there is no current consensus about treatment for apathy, and information on pharmacotherapy for this condition mainly depends upon underlying etiology and background disease. For example, dopamine agonists appear to be promising for ameliorating apathy in patients with Parkinson's disease while atypical antipsychotics used in schizophrenia and cholinesterase inhibitors have been reported to be useful for treating apathy in Alzheimer's disease and other dementias. Therefore, the treatment of apathy should be selected according to its etiology. Depressed patients with apathy should be given antidepressants, which may also alleviate other symptoms. However, caution has been raised about using SSRIs for depressed elderly persons because it may worsen apathy [58]. Since frontal lobe dysfunction is considered to be one of the causes of apathy, patients with primary apathy may respond to psychostimulants such as methylphenidate or dextroamphetamine. There have also been reports about improvement of apathy and cognitive function after stroke by treatment with cilostazol [59]. As nonpharmacological methods, cranial electrotherapy stimulation for apathy after traumatic brain injury [60], and cognitive stimulation therapy for neuropsychiatric symptoms in Alzheimer's disease [61] might have some value, but evidence awaits future studies. 

Apathy syndrome is associated with many diseases, but whether medications are applicable across this spectrum of background diseases remains unknown. For example, would cholinesterase inhibitors that are used in patients with Alzheimer's disease be effective for apathy associated with major depression? These issues should be examined in future studies.

## Figures and Tables

**Table 1:** Diagnostic criteria for apathy.

Lack of motivation relative to the patient's previous level of functioning or the standards of his or her age and culture,
as indicated either by subjective account or observation by others. Presence, with lack of motivation, of at least one
symptom belonging to each of the following three domains.
(i) Diminished goal-directed behavior:
(a) lack of effort,
(b) dependency on others to structure activity.
(ii) Diminished goal-directed cognition:
(a) lack of interest in learning new things or in new experiences,
(b) lack of concern about one's personal problems.
(iii) Diminished emotion:
(a) unchanging affect,
(b) lack of emotional responsivity to positive or negative events.
The symptoms cause clinically significant distress or impairment in social, occupational, or other important areas of
functioning. The symptoms are not due to a diminished level of consciousness or the direct physiological effects of a
substance (e.g., a drug of abuse, a medication).

Adapted from Starkstein [30].

**Table 2:** Possible medications for apathy.

Category	Class	Main background disease	Representative drug name
Antidepressants	SSRIs* SNRIs** NaSSAs*** DNRIs**** Tetracyclic antidepressants Tricyclic antidepressants	Depression	Fluvoxamine, Paroxetine Sertraline Milnacipran Mirtazapine Bupropion Maprotiline Amoxapine Nortriptyline

Dopamine stimulants	Dopamine agonists	Parkinson's disease, depression (?)	Bromocriptine Pramipexrole Ropinirole Amantadine
	MAO-B inhibitor		Selegiline

Antipsychotic agents	Atypical antipsychotic agents	Negative symptoms (apathy-like symptoms) of schizophrenia	Clozapine, Risperidone, Olanzapine, Quetiapine, Ziprasidone

Psychostimulants	Dopaminergic agents	Primary apathy or apathy syndrome	Methylphenidate Pemoline Amphetamine Modafinil

Antidementia agents	Cholinesterase inhibitors	Alzheimer's disease	Donepezil Galantamine Rivastigmine Metrifonate Tacrine
	Pyrrolidone-type nootropic agent	Stroke, Alzheimer's disease	Nefiracetam

Cerebral circulation and metabolism stimulants	Ergot alkaloid	Stroke	Nicergoline
Antiplatelet drugs	Phosphodiesterase inhibitor		Cilostazol

*Selective serotonin reuptake inhibitors: there have been a few reports that SSRIs are not effective for apathy. **Serotonin-noradrenaline reuptake inhibitors. ***Noradrenergic and specific serotonergic antidepressants. ****Noradrenaline-dopamine reuptake inhibitors.
